# Supplementary material for: Empirical methods for controlling false positives and estimating confidence in ChIP-Seq peaks
Source: BMC Bioinformatics. 2008 Dec 5;9:523. doi: 10.1186/1471-2105-9-523 (PMC2628906; doi:10.1186/1471-2105-9-523)
Supplement: Additional File 1 — A variety of html documents from the USeq web site detailing the available applications, their best usage, output file type descriptions, command line menus, etc. [file 1471-2105-9-523-S1.zip › USeqUserGuides/JavaDoc.zip!/JavaDoc/edu/utah/seq/data/HeatMapMakerPosNeg.html]

HeatMapMakerPosNeg


|  |  |  |
| --- | --- | --- |
| **Overview** \| **Package** \| **Class** \| **Use** \| **Tree** \| **Deprecated** \| **Index** \| **Help** | |  |
| **PREV CLASS**   **NEXT CLASS** | **FRAMES**    **NO FRAMES**     **All Classes** |  |
| SUMMARY: NESTED \| FIELD \| CONSTR \| METHOD | DETAIL: FIELD \| CONSTR \| METHOD |  |


---


## edu.utah.seq.data Class HeatMapMakerPosNeg

```
java.lang.Object
  edu.utah.seq.data.HeatMapMakerPosNeg
```

---

``` public class HeatMapMakerPosNeg extends java.lang.Object ```

Converts a SmoothingWindow[] into a heat map specific bar file for import into IGB.
Assumes windows are sorted by start position
Score index is the index to use when fetching a score for a window from it's float[] of scores.
Note, this will place at the 0 and 1 base two values to maintain symmetry in the values.

---

**Constructor Summary**

`HeatMapMakerPosNeg(int scoreIndex,
float minWindowFilter,
float maxWindowFilter)`


| **Method Summary** | |
| --- | --- |
| `void` | `add(int base, float value)`             Adds a line to the global ArrayLists. |
| `void` | `addHeatMapBlocks(int startIndex, int stopIndex)`             Make a heatmap blocks from given window indexes, stop not included. |
| `void` | `assembleHeatMapBlocks()` |
| `void` | `balanceValues()` |
| `SmoothingWindow[]` | `filterWindows(SmoothingWindow[] windows)`             Tosses any windows within the min max filter range. |
| `float` | `getMaxWindowFilter()` |
| `double` | `getMinWindowFilter()` |
| `PointData` | `makeHeatMapPositionValues(SmoothingWindow[] win)`             Makes a stair step heat map from an array of windows in bar format. |
| `boolean` | `overlapOrAbut(SmoothingWindow left, SmoothingWindow right)`             Assumes right window is to right of left or abuts or doesn't overlap. |
| `void` | `setMaxWindowFilter(float maxWindowFilter)` |
| `void` | `setMinWindowFilter(float minWindowFilter)` |

**Methods inherited from class java.lang.Object**

`equals, getClass, hashCode, notify, notifyAll, toString, wait, wait, wait`

**Constructor Detail**

### HeatMapMakerPosNeg

```
public HeatMapMakerPosNeg(int scoreIndex,
                          float minWindowFilter,
                          float maxWindowFilter)
```


**Method Detail**

### filterWindows

```
public SmoothingWindow[] filterWindows(SmoothingWindow[] windows)
```

:   Tosses any windows within the min max filter range.

---


### makeHeatMapPositionValues

```
public PointData makeHeatMapPositionValues(SmoothingWindow[] win)
```

:   Makes a stair step heat map from an array of windows in bar format.
    Don't forget to set the barDirectory and score Index!!!!!!!

---


### balanceValues

```
public void balanceValues()
```

---


### assembleHeatMapBlocks

```
public void assembleHeatMapBlocks()
```

---


### addHeatMapBlocks

```
public void addHeatMapBlocks(int startIndex,
                             int stopIndex)
```

:   Make a heatmap blocks from given window indexes, stop not included.

---


### add

```
public void add(int base,
                float value)
```

:   Adds a line to the global ArrayLists.

---


### overlapOrAbut

```
public boolean overlapOrAbut(SmoothingWindow left,
                             SmoothingWindow right)
```

:   Assumes right window is to right of left or abuts or doesn't overlap.

---


### getMaxWindowFilter

```
public float getMaxWindowFilter()
```

---


### setMaxWindowFilter

```
public void setMaxWindowFilter(float maxWindowFilter)
```

---


### getMinWindowFilter

```
public double getMinWindowFilter()
```

---


### setMinWindowFilter

```
public void setMinWindowFilter(float minWindowFilter)
```


---


|  |  |  |
| --- | --- | --- |
| **Overview** \| **Package** \| **Class** \| **Use** \| **Tree** \| **Deprecated** \| **Index** \| **Help** | |  |
| **PREV CLASS**   **NEXT CLASS** | **FRAMES**    **NO FRAMES**     **All Classes** |  |
| SUMMARY: NESTED \| FIELD \| CONSTR \| METHOD | DETAIL: FIELD \| CONSTR \| METHOD |  |


---
